# The role of cyano-phycocyanin as a quorum sensing inhibitor to attenuate *Pseudomonas aeruginosa* virulence

**DOI:** 10.3389/fcimb.2025.1624927

**Published:** 2025-11-19

**Authors:** Jun Ying, Shiqun Guo, Yunru Pan, Dong Li, Yijia Tai, Yue Li, Qinyu Lin, Lingling Pan, Dadao Huang, Hanqi Xiao, Ruowang Pan, Xiaomin Xu, Jing Xie, Zhefeng Lou, Peizhen Li

**Affiliations:** 1 School of Laboratory Medicine and Life Science, Wenzhou Medical University, Wenzhou, China; 2 Wenzhou Semir United International School, Wenzhou, China; 3 Rehabilitation Medicine, No.906 Hospital of Joint Logistic Support Force of PLA, Wenzhou, China; 4 Wenzhou Third Clinical Institute Affiliated to Wenzhou Medical University, Wenzhou People’s Hospital, Wenzhou, China

**Keywords:** C-PC, PQS, *Pseudomonas aeruginosa*, biofilm, RAW264.7

## Abstract

**Introduction:**

In recent years, developing drugs that directly target pathways related to the pathogenic mechanisms of bacteria has been a research hotspot, and quorum sensing (QS) is one of the most effective strategies to combat multidrug-resistant bacteria. We evaluated the inhibitory effect of cyano-phycocyanin (C-PC) on the virulence of PA2 *in vitro* and *in vivo* and explored its potential as a quorum sensing inhibitor (QSI).

**Methods:**

In this study, the minimum inhibitory concentration (MIC) and growth curve of C-PC on PA2 were determined, and the effect of C-PC on QS-regulated virulence factors and the anti-biofilm activity of C-PC against PA2 were investigated. The *Pseudomonas* quinolone signal (PQS) were analyzed by HPLC system and the expression levels of QS signaling molecules, virulence genes, biofilm and movement-related genes were detected by qPCR. The macrophage and mouse infection model were used to explore the anti-infection ability of C-PC *in vitro* and *in vivo*.

**Results:**

C-PC attenuated biofilm formation, pyocyanin synthesis, motility, and PQS signaling molecule production. Moreover, C-PC significantly downregulated the transcription levels of QS-related genes in PA2, including *pqsA* and *pqsR*, as well as the expression of virulence factor genes *phzA*, *lasA*, *lasB*, *flgF*, *fliE*, *exoS*, *exsA*, *lecA*, *popB*, *vasG*, *chiC*, *pelF*, *pslB*, *qseB*, and *pgsR*. *In vitro*, C-PC reduced the adhesion and invasion of PA2 in RAW264.7 cells, and decreased LDH release and macrophage damage caused by PA2 infection. *In vivo*, C-PC reduced the pathogenicity of PA2 and improved mouse survival rates, showing a protective effect.

**Conclusion:**

Taken together, these results suggest that C-PC showed strong anti-QS activity, providing new insights into the development of strategies against PA infection.

## 1 Introduction

*Pseudomonas aeruginosa* (PA) is a ubiquitous Gram-negative opportunistic pathogen often isolated from plants, fruits, soil, and water environments (Sousa and Pereira, 2014). Under certain circumstances, PA can be a significant pathogenic factor in severe opportunistic infections in humans and animals (Capatina et al., 2022). It typically infects the airways and urinary tract, causes blood stream infections, and is the most common cause of burn wound infections, hot-tub dermatitis, and outer ear infections (Szabó et al., 2022). Due to its strong survival adaptability and high susceptibility to drug resistance, complete eradication is challenging. Different degrees of resistance have emerged against the five commonly used clinical antibiotics, particularly imipenem and meropenem, with resistance rates of 23% and 18.9%, respectively (Ying et al., 2017; Chang et al., 2018). Additionally, PA is one of the six highly drug-resistant pathogens in the “ESKAPE” (*Enterococcus faecalis*, *Staphylococcus aureus*, *Klebsiella pneumoniae*, *Acinetobacter baumannii*, *Pseudomonas aeruginosa*, *Escherichia coli*) group, which are major contributors to hospital-acquired infections (World Health Organization, 2017). Thus, the World Health Organization (WHO) has classified carbapenem-resistant PA as a key priority pathogen urgently requiring new antibiotics. Finding alternative therapies to eliminate PA infections have become crucial (Jurado-Martín et al., 2021).

The mechanism of traditional antibiotics is to interfere with the normal physiological metabolism of bacteria, exerting antibacterial and bactericidal effects and even playing a targeted screening role in bacterial resistance mutations. It has led to increasingly severe antibiotic resistance (Liu et al., 2018). In recent years, developing drugs that directly target pathways related to the pathogenic mechanisms of bacteria has been a research hotspot (Sanya et al., 2023). The virulence of PA plays an important role in its pathogenicity. The expression and regulation of virulence genes are closely related to bacterial quorum sensing (QS) (Lee and Zhang, 2015; Vadakkan et al., 2024). PA has a complex QS regulatory network, consisting of four QS systems: the *LasI/LasR* system, *RhlI/RhlR* system, IQS system, and PQS system (Meng et al., 2020). These systems coordinate and regulate the expression of over 300 genes involved in virulence, affecting motility, virulence factor production, biofilm formation, and antibiotic resistance (Vadakkan et al., 2024). Anti-virulence drugs targeting the QS system can block the perception and transduction of self-inducers, effectively reducing the pathogenicity of PA without threatening the survival of bacteria. This approach prevents the development of drug resistance and shows great potential for the research of anti-infective therapies (Grossman et al., 2020).

The behavior of bacteria is characterized by mutual assistance with coexisting organisms and competition with other organisms, enabling the screening of numerous quorum sensing inhibitors (QSIs) in nature (Defoirdt, 2018). Among them, marine algae are a rich source for drug discovery. The active ingredient in *Spirulina platensis*, C-PC accounts for 40% of the total protein and has physiological activities such as antioxidant, anticancer, anti-inflammatory, immunomodulatory, and antibacterial properties (Bannu et al., 2019; Pan et al., 2015). However, the potential mechanism by which C-PC regulates virulence and inflammation to reduce pathological damage in PA infection has not yet been fully studied *in vitro* and *in vivo*. In this context, we further examined the effect of C-PC on QS-related phenotypes, including biofilm formation and virulence factors. We also investigated the anti-infective activity and mechanism of action of C-PC on the PA^’^s QS system at the gene level. These results suggest C-PC as a promising anti-QS agent that may help prevent PA-mediated inflammation and infection.

## 2 Materials and methods

### 2.1 C-PC extraction

C-PC was extracted from *Spirulina platensis* and isolated using ammonium sulphate precipitation, followed by a single-step chromatography process with DEAE Cellulose-52 and phosphate buffer (Pan et al., 2015). As a result, the high-purity C-PC (with an A_620_/A_280_ value of 4.36) was obtained.

### 2.2 Bacterial strains and cell strains

PAO1 (ATCC 15692), and PA2 (collected from the First Affiliated Hospital of Wenzhou Medical College) were stored in our laboratory. The strains were grown in Luria Bertani (LB) medium at 37°C with shaking, collected by centrifugation, and diluted in the appropriate experimental medium.

RAW264.7 cells were purchased from the Institute of Biochemistry and Cell Biology Sciences, Chinese Academy of Sciences (Shanghai, China). Cells were cultured in DMEM medium (Gibco, New York, USA) supplemented with 10% fetal bovine serum (Chinese Holly Biotechnology Ltd., Hangzhou, China).

### 2.3 Determination of minimum inhibitory concentration

The MIC of C-PC against PA was determined using the broth microdilution method following the guidelines of the Clinical and Laboratory Standards Institute (Gao et al., 2022). Briefly, 5 × 10^6^ colony-forming units (CFU)/mL of PA were dispensed into LB medium supplemented with 0, 16, 32, 64, 128, 256, 512, and 1024 μg/mL of C-PC or Ampicillin (AMP) (Yuanye, Shanghai, China). The plates were incubated at 37 °C for 24 h.

### 2.4 Growth curve

Briefly, 5 × 10^6^ CFU/mL of PA2 was diluted in LB medium cultured with different concentrations of C-PC (0, 64, 128, 256 and 512 μg/mL) at 37°C with continuous shaking (180 rpm). Every 2 h, the absorbance of each bacterial culture was measured at 600 nm using a microplate reader.

### 2.5 Quantitation of pyocyanin

This experiment was performed as above in 2.4 and cultured for 12 h. For cell-free quantification of virulence factors, pyocyanin was extracted from the culture supernatant using chloroform (800 µL) at a 3:2 ratio, followed by re-extraction with 200 µL of 0.2 mol/L HCl (Yang et al., 2021). Sterilized deionized water was used in negative control. The absorbance was measured at 520 nm.

### 2.6 Swimming and swarming motility assays

The plates were prepared according to an established protocol (Rashid and Kornberg, 2000). PA2 was cultured with C-PC (0, 64, 128, and 256 µg/mL) for 24 h. Next, 1.0 μL of PA2 culture was inoculated at the center of swimming and swarming plates. The plates were incubated at 37 °C for 12 h, and the flagellar motility was determined by measuring the diameter (mm) of the traveled cells on the surface of the agar plate (Rashmi et al., 2018).

### 2.7 Biofilm inhibition assay

PA2 was cultured with different concentrations of C-PC (0, 64, 128, and 256 µg/mL) for 24 h at 37 °C. After incubation, the culture medium was removed, and the tubes were washed three times with phosphate-buffered saline (PBS). The remaining biofilms were stained with 0.1% crystal violet for 15 min and then rinsed twice with water. The tubes were dried, and the biofilm-bound crystal violet was solubilized using 95% ethanol. The absorbance was measured at 595 nm (Yu et al., 2014).

For fluorescence imaging, PA2 in the logarithmic growth phase was seeded into a 24-well plate containing coverslips and incubated in LB medium with different concentrations of C-PC (0, 64, 128, and 256 µg/mL) at 37 °C. After 24 h of incubation, the medium was removed, and the formed biofilms were carefully washed three times with PBS to remove non-adherent bacteria. Subsequently, the biofilms were stained with Calcein-AM and propidium iodide (PI) (Li et al., 2015). Following two washes with PBS, the biofilms were imaged using fluorescence microscopy (Nikon, Melville, NY).

### 2.8 Detection of PQS signaling molecules

*Pseudomonas* quinolone signal (2-heptyl-3-hydroxy-4-quinolone, PQS) was extracted using acidified ethyl acetate (0.5% formic acid, vol/vol) and analyzed by an Accela HPLC system (Thermo Fisher Scientific,Waltham, MA, USA) (Xu et al., 2016). The assay was performed using a Zorbax Eclipse XDB-C_18_ column (5 μm, 4.6 mm×250 mm, Agilent, Santa Clara, CA, USA). The PQS was eluted at a flow rate of 1 mL/min in an isocratic elution mode with a mobile phase consisting of 86% methanol (acidified with 1% glacial acetic acid) and 14% water (acidified with 1% glacial acetic acid). The PQS concentration in the samples was calculated by comparing the peak areas of a PQS standard (50 µg/mL, Sigma, USA) with those of PA2. The relative PQS production was normalized against the total bacterial protein content.

### 2.9 Determination of mRNA expression levels of QS-related virulence genes in PA2 by qPCR

Total RNA was extracted and reverse transcribed into complementary DNA (cDNA), following the instructions provided in a commercial kit (Takara Bio, Inc., Dalian, China). Quantitative polymerase chain reaction (qPCR) was performed to assess the expression of QS-related genes. **Supplementary Table S1** listed the primers used to amplify the reference genes. The relative differences in mRNA expression levels were calculated using the 2^-△△CT^ method.

### 2.10 Cell viability assay

To assess the cytotoxic influence of C-PC on macrophages, we conducted the the CCK-8 assay. RAW264.7 cells (1 × 10^5^ cells/mL) were seeded in 96-well plates and incubated at 37 °C overnight, and then treated with 0, 32, 64, 128, 256, and 512 μg/mL C-PC for 24 h. Then, 10 µL CCK-8 (Sangon Biotech, Shanghai, China) was added to each well, and the cells were incubated for 4 h. Afterwards, the absorbance of the cells at 450 nm was measured using a microplate reader.

### 2.11 Adhesion and invasion assay

RAW264.7 cells were seeded into 12-well tissue culture plates at a density of 5×10^5^ cells/well and cultured until 80-90% confluence. PA2 was prepared to a concentration of 1×10^8^ CFU/mL in DMEM without antibiotics. Cells were incubated with PA2 at three multiplicities of infection (MOIs: 25, 50, or 100) in the absence of antibiotics. After 2 or 4 h of infection, the cells were washed with PBS and lysed with 0.5% Triton-100 for 15 minutes at 37 °C. For the invasion assay, extracellular bacteria were killed by incubating with 200 µg/mL gentamicin for 1 h. The attachment and invasion levels were determined by bacterial plate count (Tang et al., 2022).

### 2.12 Lactate dehydrogenase assay

The cells were infected with PA2 (MOI = 100:1) for 4 h, followed by treatment with C-PC at concentrations of 0, 32, 64, and 128 µg/mL. The culture supernatants were collected and centrifuged at 12,000 rpm for 5 min. LDH activity was measured using an LDH cytotoxicity assay kit (Jiancheng Biotech, Nanjing, China).

### 2.13 *In vivo* protection assay

All animal study protocols were reviewed and approved by the animal welfare committee of Wenzhou Medical University (approval number: SYXK2014-0052). A mouse survival assay was performed to evaluate the protective effect of C-PC against PA2 (Khayyat et al., 2021a). Five groups of healthy albino mice (5 weeks old, with similar weight) were used in the study. Two control groups received either no bacteria or an intraperitoneal (i.p.) injection of 100 µL of sterile PBS. One positive group was injected with 100 µL of untreated PA2, while two test groups were injected with 100 µL of PA2 treated with C-PC at 1/4 MIC (256 µg/mL), and 1/8 MIC (128 µg/mL), respectively. All mice were housed under standard conditions with appropriate ventilation and feeding. The survival of mice in each group was monitored daily for five days. Tissue samples were harvested for pathological analysis (Khayyat et al., 2021b).

### 2.14 Statistical analysis

All experiments were performed in triplicate, and the results were presented as mean ± standard deviation (SD). Statistical analyses were performed using SPSS 23.0. Differences between groups were analyzed using one-way ANOVA. *P* < 0.05 was considered statistically significant.

## 3 Results

### 3.1 Antimicrobial activity

**Figure 1:**
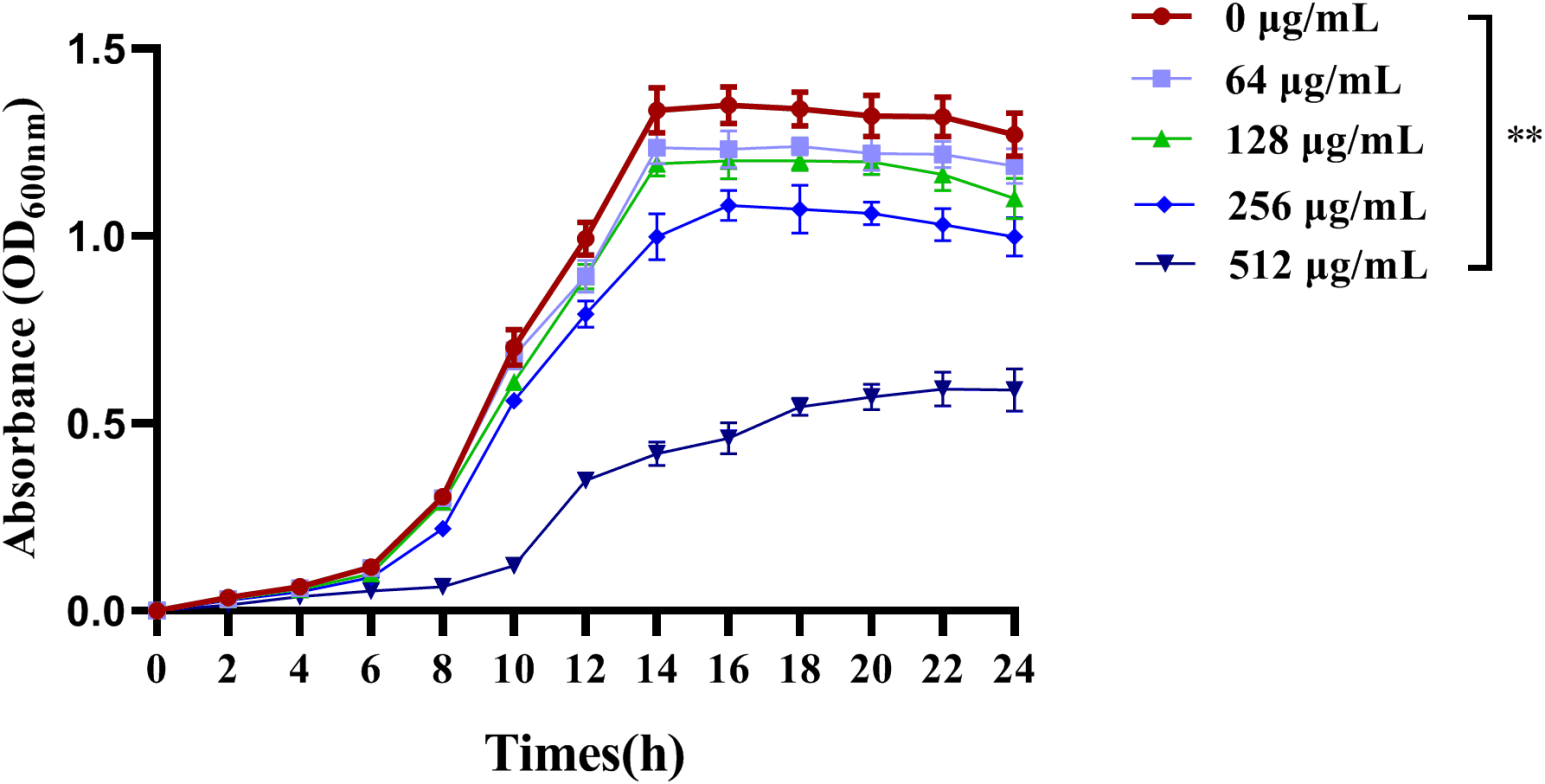
Effects of different concentrations of C-PC on the growth of PA2. All data were expressed as means ± SD (n = 3). ^**^P < 0.01 versus the untreated control group.

The antimicrobial activity of C-PC was evaluated by the MIC microdilution method. The MICs of C-PC against PAO1 and PA2 were 512 μg/mL and 1024 μg/mL, respectively. For AMP, the MICs were 512 μg/mL and 256 μg/mL. The effect of C-PC on the growth of PA2 was assessed using growth curves at sub-MIC concentrations, with no growth inhibition observed at concentrations below 1/4 times the MIC of C-PC (**Figure 1**). So the sub-MIC concentration was selected to evaluate the anti-biofilm and anti-QS activity.

### 3.2 C-PC attenuated the production of pyocyanin

**Figure 2:**
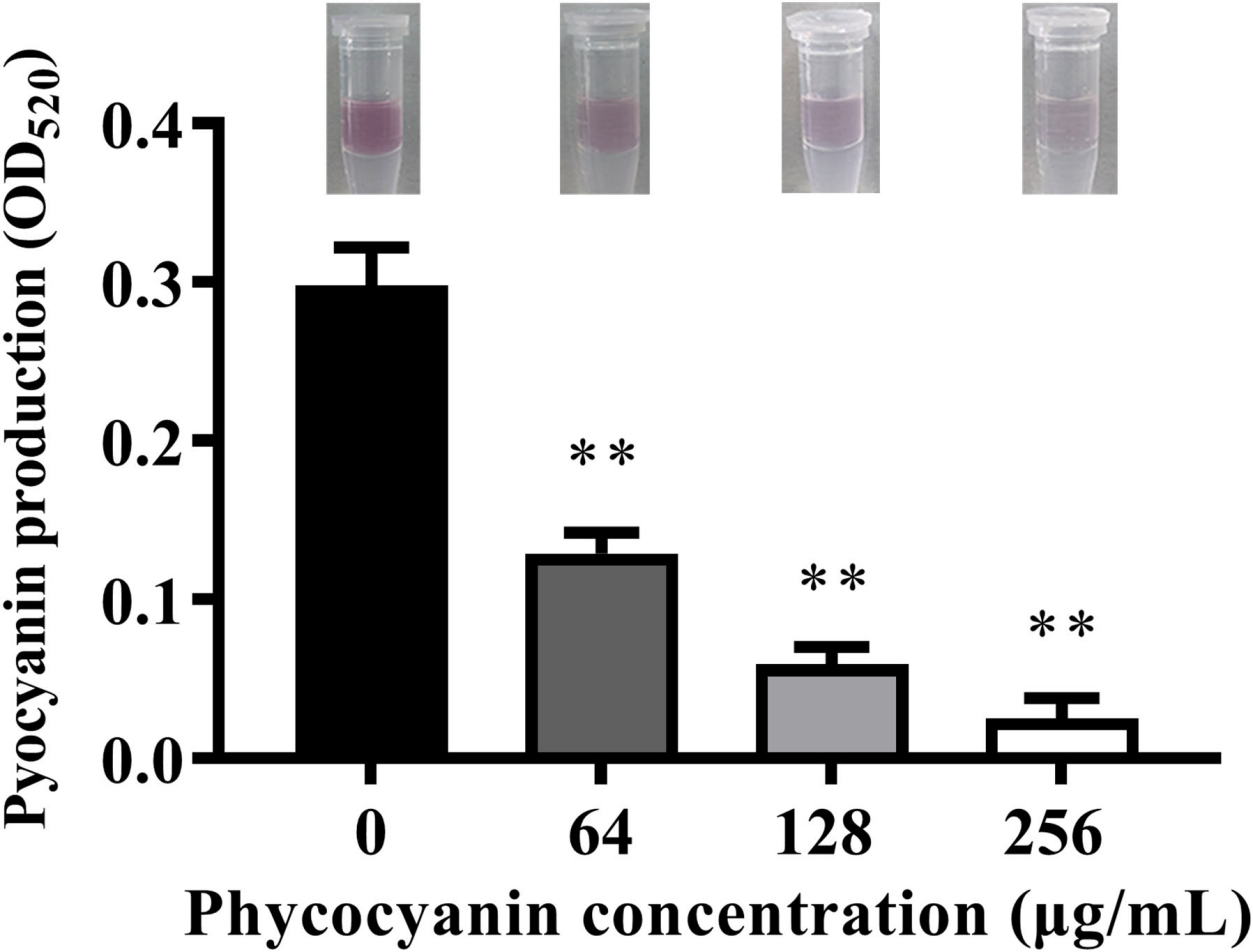
Effect of sub-MICs of C-PC on inhibition of pyocyanin production in PA2. All data were expressed as means ± SD (n = 3). ^**^P < 0.01 versus the untreated control group.

The inhibitory effect of C-PC on pyocyanin production was evaluated. As shown in **Figure 2**, pyocyanin production was significantly decreased by C-PC treatment in a concentration- dependent manner (^**^P < 0.01). C-PC at a concentration of 256 µg/mL was found to have the strongest inhibitory effect on pyocyanin.

### 3.3 C-PC inhibited the swimming and swarming motility of PA2

**Figure 3:**
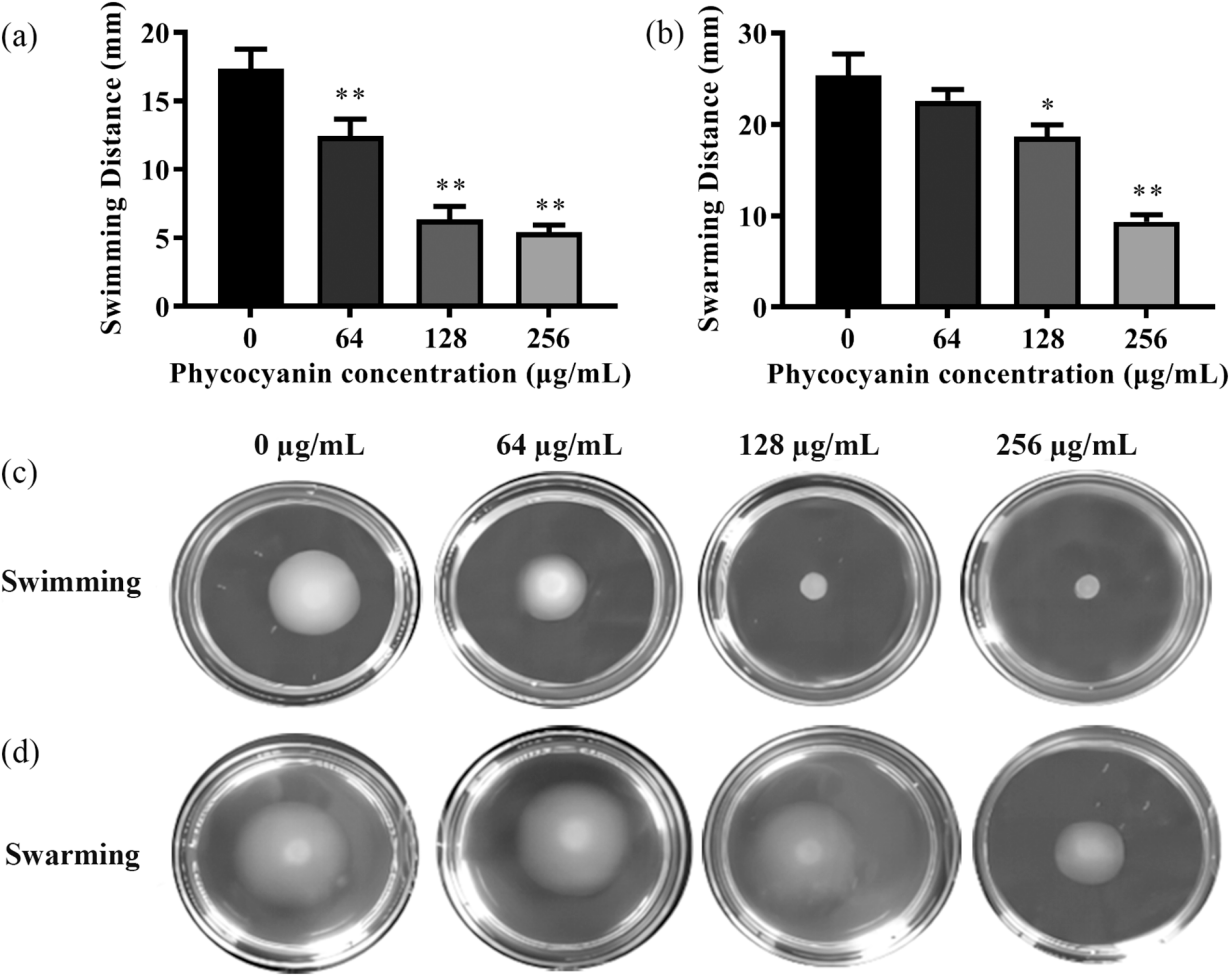
Effect of sub-MICs of C-PC on inhibition of motility in PA2. **(a, b)** The swimming and swarming diameter statistics. All data were expressed as means ± SD (n = 3). ^*^P < 0.05 and ^**^P < 0.01 versus the untreated control group. **(c)** Swimming motility. **(d)** Swarming motility.

As shown in **Figure 3**, C-PC reduced both swimming and swarming motility (^*^P < 0.05, ^**^P < 0.01). 28.21, 63.34 and 68.75% decrease in swimming motility was determined in the presence of 64, 128 and 256 µg/mL C-PC, and 26.45, 63.14% decrease in swarming motility was determined in the presence of 128, 256 µg/mL C-PC against PA2. An increase in C-PC concentration led to a significant inhibitory effect on PA2 flagellar motility.

### 3.4 C-PC inhibited biofilm formation

**Figure 4:**
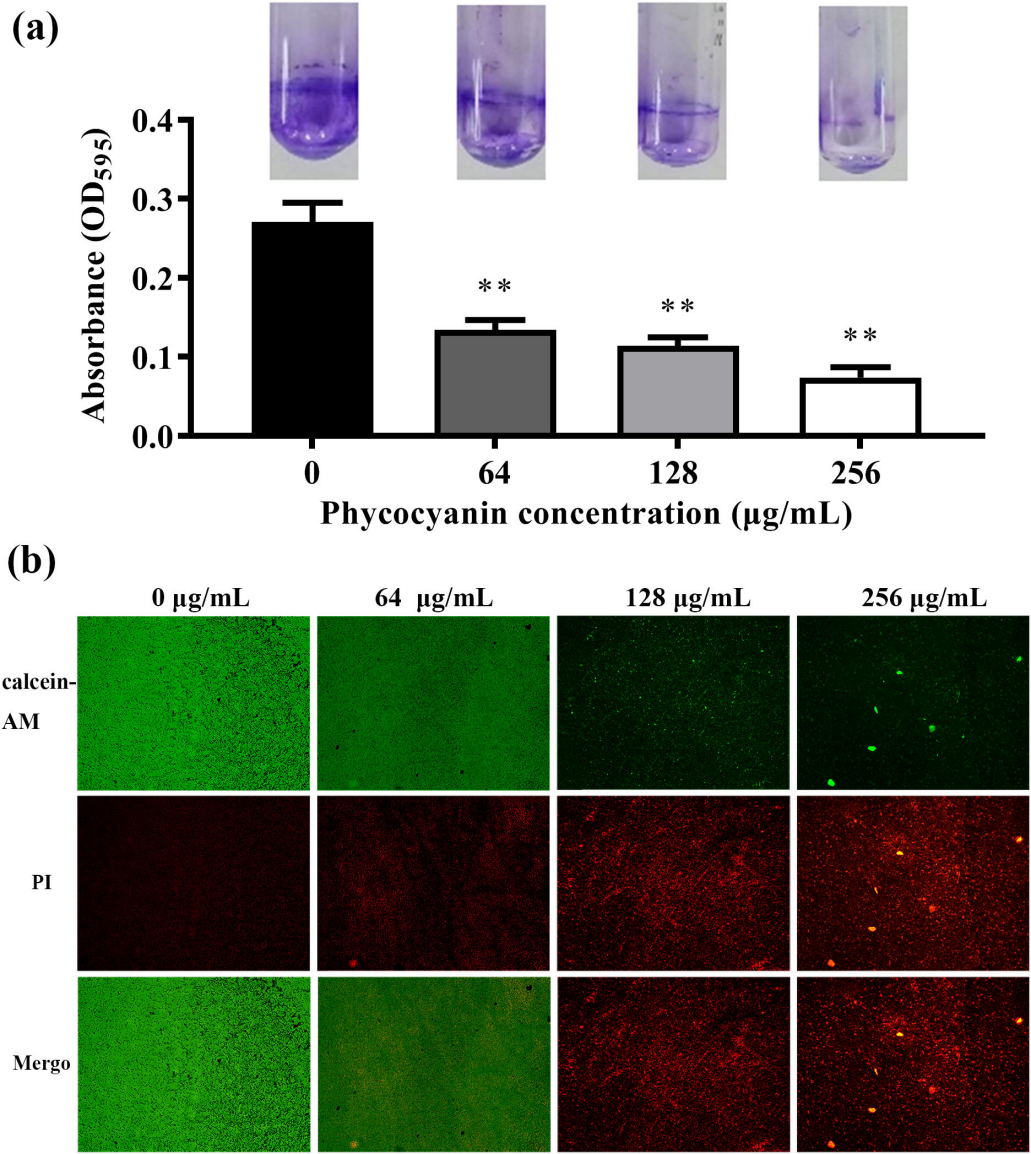
Effects of C-PC on biofilm formation of PA2 at different concentrations. **(a)** The biofilm quantified by crystal violet staining and measuring at A570 nm. **(b)** Light microscopic images. All data were expressed as means ± SD (n = 3). ^**^P < 0.01 versus the untreated control group.

C-PC significantly reduced PA2 biofilm formation at 24 h in a dose-dependent manner (^**^P < 0.01, **Figure 4A**). As shown in **Figure 4B**, untreated PA2 biofilms stained with calcein-AM emitted bright green fluorescence, indicating intact biofilms that were dense and thick. In contrast, the biofilms of the C-PC-treated group were predominantly stained with red fluorescence (PI), indicating substantial damage to the biofilm structure.

### 3.5 Inhibition of the QS system by C-PC

**Figure 5:**
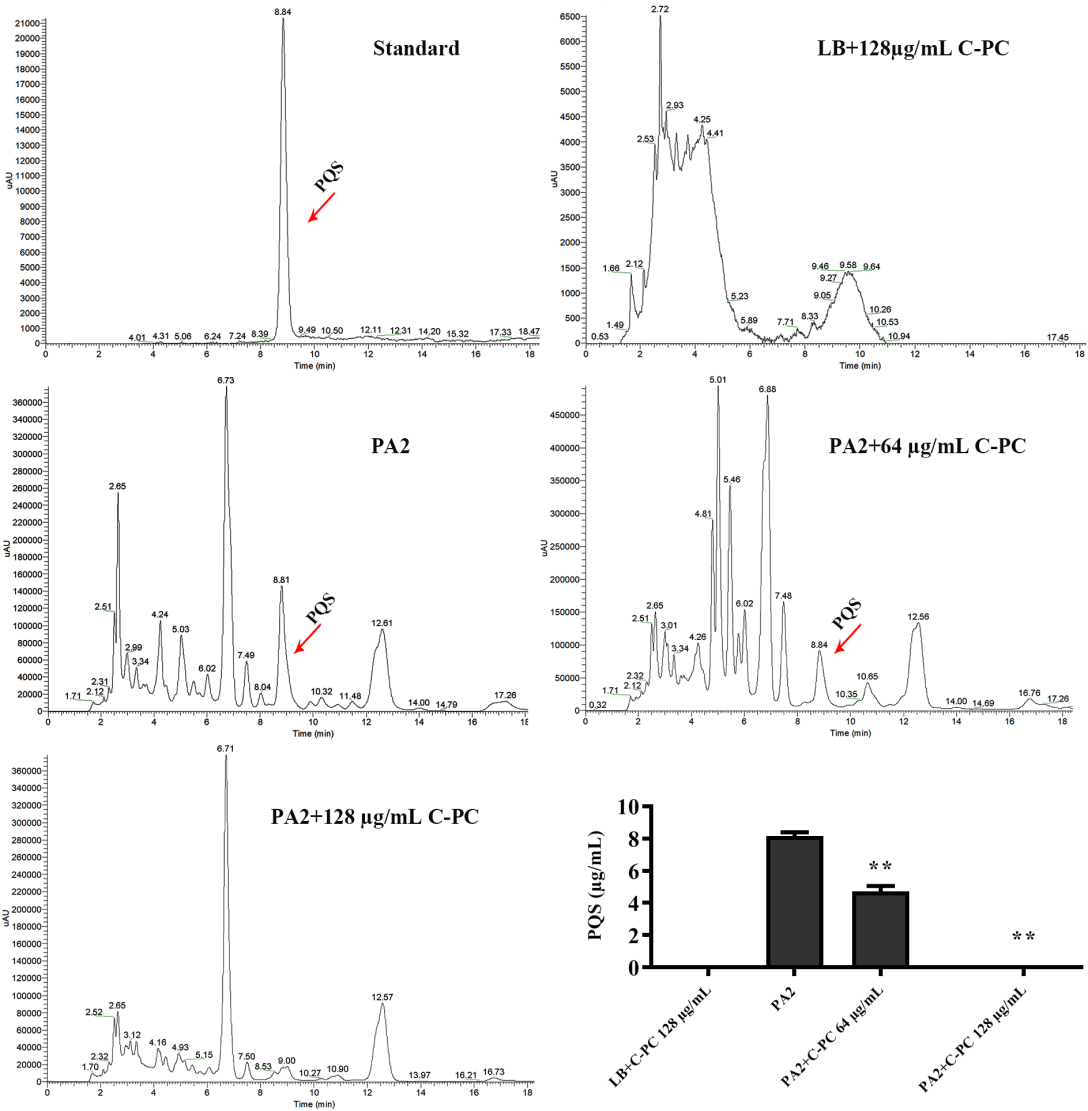
Effect of C-PC on inhibition of PQS production in PA2 analysied by HPLC assays. All data were expressed as means ± SD (n = 3). ^**^P < 0.01 versus the PA2.

**Figure 6:**
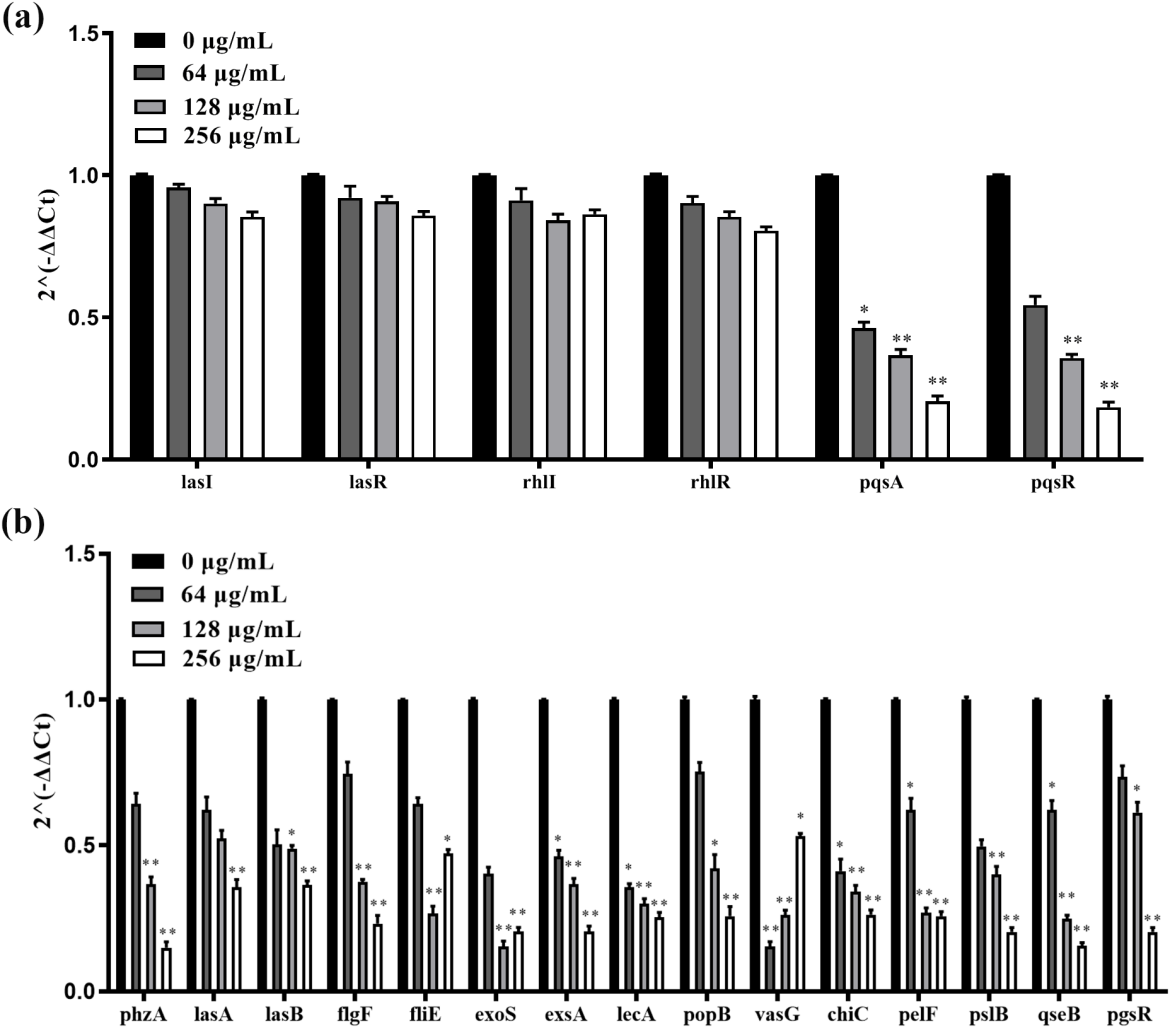
Transcriptional levels of the QS-related **(a)** and virulence genes **(b)** in PA2 with different concentrations C-PC for compared with untreated control detected by qPCR. All data were expressed as means ± SD (n = 3). ^*^P < 0.05, ^**^P < 0.01 versus the untreated control group.

The effect of C-PC on the synthesis of signaling molecules in PA2 was assessed using HPLC analysis. As shown in **Figure 5**, compared with PA2, the PQS yield in cultures exposed to C-PC was significantly decreased (^**^P < 0.01). C-PC at a concentration of 128 µg/mL was found to have the strongest inhibitory effect. Additionally, the expression of QS-related genes, including *lasI*, *lasR*, *rhlI*, *rhlR*, *pqsA*, and *pqsR*, were analyzed. The results showed that the expression of key genes in the PQS system, including *pqsA* and *pqsR*, was downregulated by C-PC treatment (^**^P < 0.01, **Figure 6A**). And the expression of other key QS system genes, such as *rhlI/R* of the rhl system and *lasI/lasR* of the las system, remained unchanged.

### 3.6 Viability and cytotoxicity of C-PC on macrophages infected with PA2

**Figure 7:**
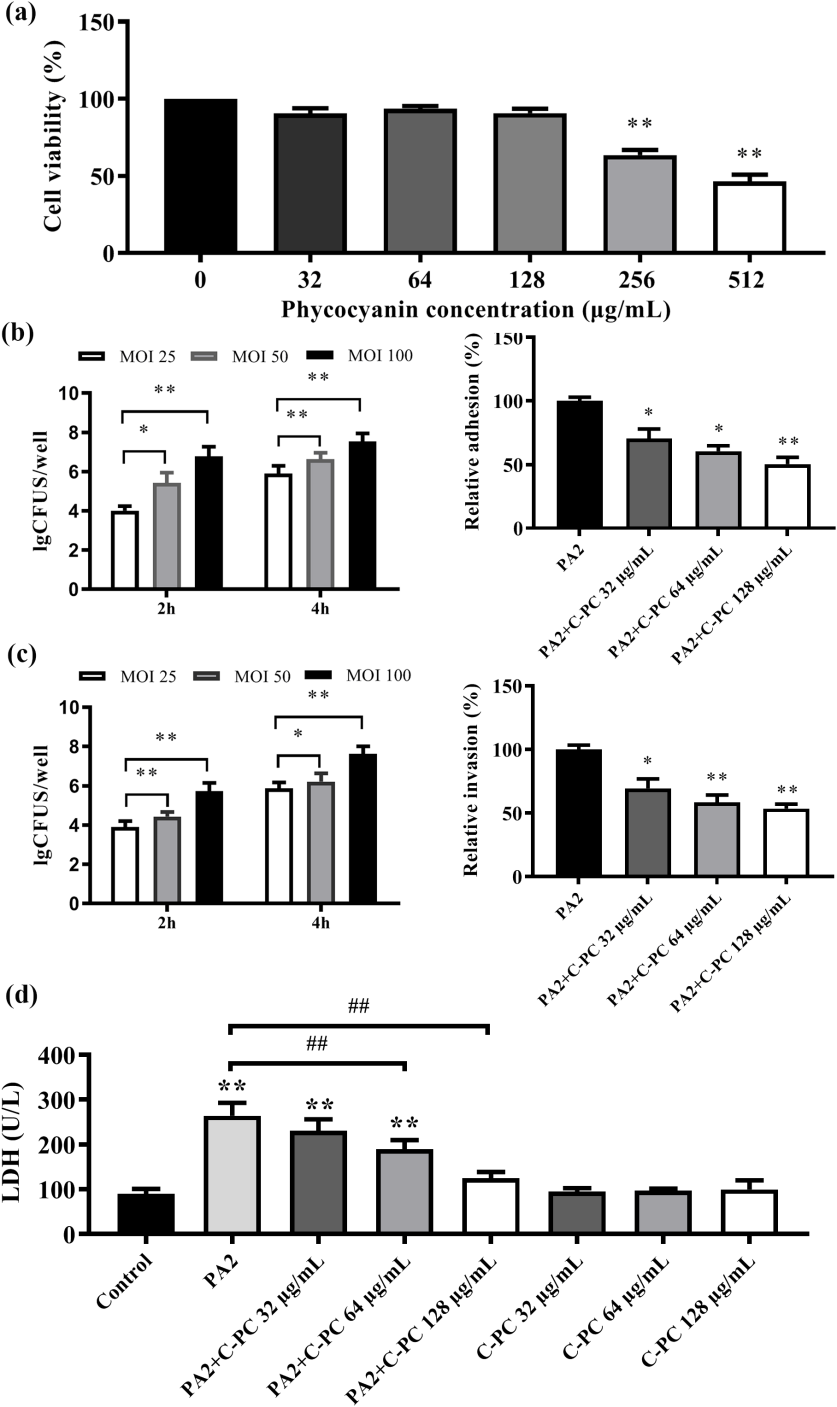
Effects of C-PC on adhesion and invasion of PA2 infected RAW264.7 cells. **(a)** Effect of different concentrations of C-PC on RAW264.7 cell viability activity. **(b)** The adhesion of PA2 infected RAW264.7 cells. **(c)** The invasion of PA2 infected RAW264.7 cells. **(d)** Effects of C-PC on LDH activity of RAW264.7 cells infected with PA2. All data were expressed as means ± SD (n = 3). *P < 0.05, **P < 0.01 versus the untreated control group. ##P < 0.01 versus the PA2 group.

To determine the experimental concentrations of C-PC for RAW264.7 cells in subsequent experiments, the CCK-8 experiment was used to evaluate the cytotoxic effects of C-PC. The initial concentrations of C-PC were chosen according to previously established and published protocols (Zhang et al., 2022). As depicted in **Figure 7A**, the viability of cells remained above 90% at concentrations of 0-128 µg/mL, indicating that C-PC was not cytotoxic to RAW264.7 cells. In the infection experiment, PA2 was able to adhere to and invade the cells. As the MOI and infection time increased, the number of PA2 bacteria attaching to and invading macrophages also increased (^*^P < 0.05, ^**^P < 0.01; **Figures 7B, C**). And compared to the PA2 group, bacteria adhesion to macrophages significantly decreased after C-PC treatment. Further analysis revealed that C-PC reduced the invasion of PA2 into RAW264.7 cells. Bacterial infection leads to membrane damage, resulting in the release of LDH from the cytoplasm into the culture medium. Compared to the control group (RAW264.7), the release of LDH was significantly higher in the PA2-infected group (^**^P < 0.01). In addition, the release of LDH decreased in a concentration-dependent manner upon C-PC treatment, with no effect on macrophage proliferation at the experimental concentrations (^##^*P* < 0.01; **Figure 7D**).

### 3.7 Anti-infection activity of C-PC *in vivo*

**Figure 8:**
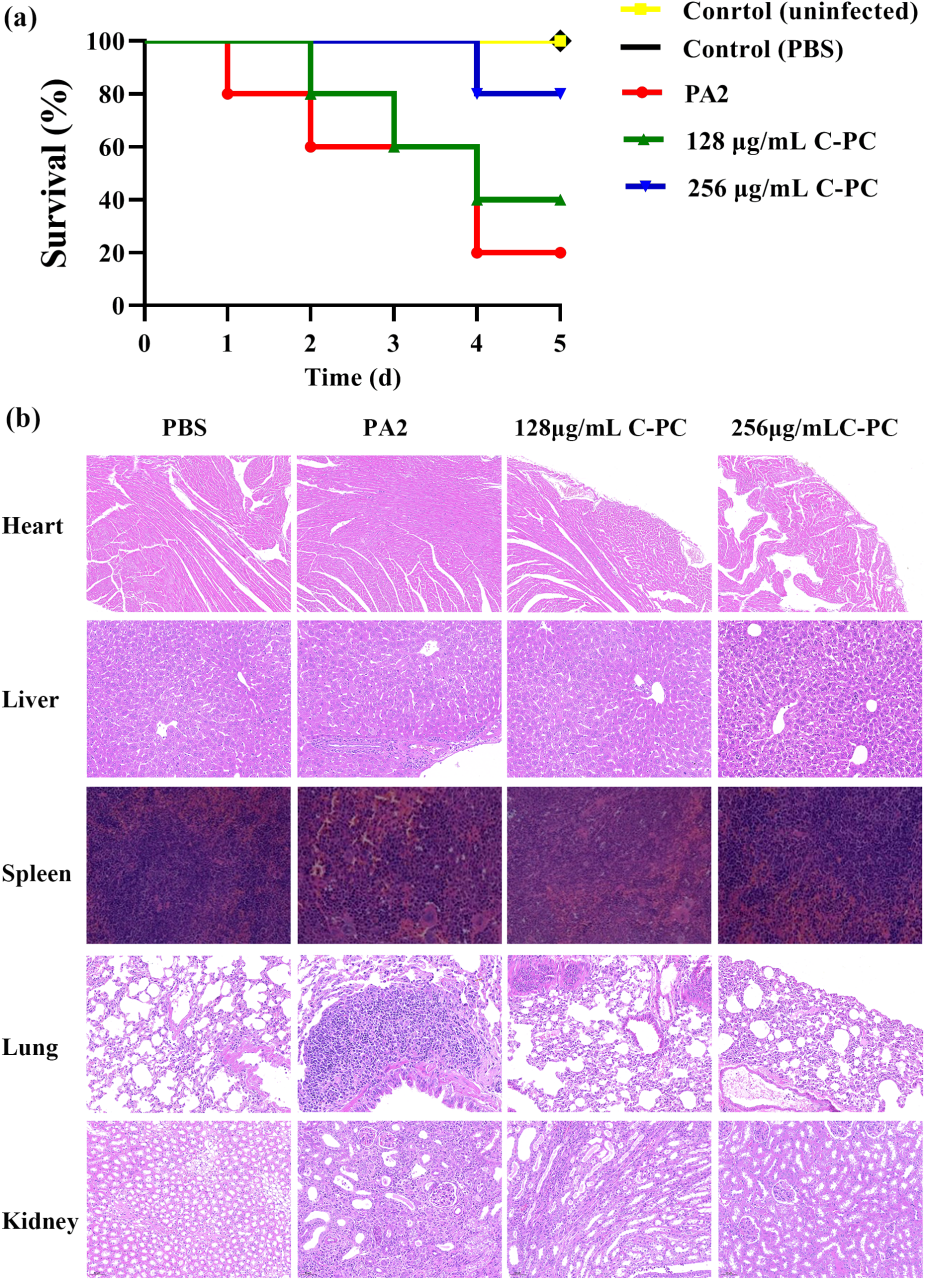
Effects of C-PC on the virulence of PA2 in a mouse model. **(a)** Mouse survival rate. **(b)** H&E staining of the heart, liver, spleen, lung, and kidney tissues.

The *in vivo* protective effects of C-PC at sub-MIC concentrations against PA2 were evaluated in five mouse groups (**Figure 8A**). No deaths were observed in the control groups, and one mouse out of five survived in the group injected with untreated PA2. In contrast, two mice out of five survived in the 1/4 MIC group, and four mice out of five survived in the 1/8 MIC group. After 5 days of infection, histological analysis using Hematoxylin-eosin (H&E) staining revealed significantly reduced infiltration of inflammatory cells in the lung, kidney and liver of mice treated with C-PC compared to the untreated PA2 group (**Figure 8B**). In summary, C-PC at sub-MIC concentrations significantly protected mice from PA infection.

## 4 Discussion

The development of antibiotic resistance among microorganisms, particularly bacteria, is a major global health challenge. In this context, inhibition of QS communication in bacteria offers a promising alternative strategy to combat multi-drug-resistant strains (Markus et al., 2023). To address bacterial infections, it is essential to discover or modify anti-QS compounds. Previous studies have shown that PC from Oscillatoria minima (16 µg/mL) exhibits inhibitory effects against bacteria such as *Pseudomonas fragi*, *Escherichia coli*, *Pseudomonas vulgaris*, *Bacillus subtilis*, *Klebsiella oxytoca*, *and Streptococcus pyogenes* (Venugopal et al., 2020). Osman A. et al. performed an *in vitro* study revealing that C-PC from *Anabaena* demonstrated antibacterial activity comparable to benzylpenicillin against Gram-positive bacteria (Dranseikienė et al., 2022). Nihal B. et al. investigated the antimicrobial activity of C-PC extracted from *Spirulina* against *Propionibacterium acne* and *Staphylococcus epidermidis* (Nihal et al., 2018). In the present study, we further explored the anti-QS potential of C-PC isolated from *Spirulina platensis*. Our results demonstrated that C-PC effectively inhibited QS in PA2, with MICs of 1024 μg/mL. Furthermore, C-PC significantly reduced biofilm formation, swimming, swarming, and pyocyanin production at concentrations of 64, 128, and 256 μg/mL, without influencing bacterial growth.

The QS system of PA has been extensively studied and is considered a promising target for developing antimicrobial drugs against this pathogen (Saeki et al., 2020). The PQS is the third QS system in PA, utilizing 2-heptyl-3- hydroxy-4-quinolone (PQS) and its biosynthetic precursor 2-heptyl-4-hydroxyquinoline (HHQ). PQS interacts with the two acyl-homoserine lactone (AHL) systems, establishing a regulatory link between these two AHL-based QS systems (Schertzer et al., 2010; Dulcey et al., 2013). The biosynthesis of PQS in PA requires proteins encoded by the pqsABCDE genes, which are controlled by the transcriptional activator PqsR (Chatterjee et al., 2020). When the concentration of PQS reaches a certain threshold, PqsR is activated, inducing the production of virulence factors such as pyocyanin (García-Reyes et al., 2020) and biofilm (Allesen-Holm et al., 2006), as well as driving the expression of *pqsA*, the first gene in the PQS biosynthetic operon (Lee and Zhang, 2015). In addition, PQS-deficient PA mutants form less biofilm, and PQS is produced in high quantities in the sputum of patients with cystic fibrosis (Rather et al., 2021). These findings provide a strong rationale for targeting PQS regulation in drug discovery efforts. In this study, the level of PQS was significantly lower in cultures exposed to C-PC. Moreover, the qPCR results showed that the two key genes *pqsA* and *pqsR* of the PQS system were down-regulated. These observations suggest that C-PC does not drastically affect Las and Rhl signaling but has a negative impact on PQS signaling. However, further studies are required to elucidate the specific targets and mechanisms involved.

Given the essential role of QS in bacterial pathogenicity and virulence, it is considered a novel and promising therapeutic strategy for combating bacterial infections and antibiotic resistance. For example, the leaf extract of Cuphea carthagensis (Jacq.) J. F. Macbr has been shown to inhibit biofilm formation and the QS system of PA at sub-MIC concentrations (Rather et al., 2021). PQS has been detected in the lungs of cystic fibrosis patients, indicating the important role of this molecule in the long-term persistence of PA infections (Abdalla et al., 2017). Antivirulence drugs can also prevent bacteria from adhering to and invading intestinal epithelial cells without affecting bacterial growth (Lories et al., 2020; Ahmad et al., 2016; Motta et al., 2021). Thus, the immune system could eliminate bacteria and reduce intestinal infection and damage. In our study, we found significant inhibition of virulence-associated genes (*phzA*, *lasA*, *lasB*, *flgF*, *fliE*, *exoS*, *exsA*, *lecA*, *popB*, *vasG*, *chiC*, *pelF*, *pslB*, *qseB*, and *pgsR*, **Figure 6B**). And, the potential therapeutic effect of C-PC was demonstrated by its ability to significantly reduce the adhesion and invasion of PA2 in RAW264.7 cells. Furthermore, mice infected with PA2 and treated with C-PC also showed significantly improved survival. Collectively, these findings suggest that C-PC may represent a promising new anti-QS and anti-inflammatory agent, warranting further investigation into the underlying mechanisms.

## 5 Conclusions

Targeting bacterial QS is a promising strategy to conquer bacterial pathogenesis, mainly, it is less likely to result in the emergence of bacterial resistance. C-PC attenuated biofilm formation, pyocyanin synthesis, motility, and PQS signaling molecule production. Furthermore, C-PC reduced the adhesion and invasion of PA2 in RAW264.7 cells and protected mice from PA2 *in vivo*. This study suggest that C-PC shows strong anti-QS activity, providing new insights into the development of strategies against PA infection.

## Data Availability

The original contributions presented in the study are included in the article/**Supplementary Material**. Further inquiries can be directed to the corresponding authors.

## References

[B1] Abdalla M. Y. Hoke T. Seravalli J. Switzer B. L. Bavitz M. Fliege J. D. . (2017). Pseudomonas quinolone signal induces oxidative stress and inhibits heme oxygenase-1 expression in lung epithelial cells. Infect. Immun. 85, e00176–e00117. doi: 10.1128/IAI.00176-17, PMID: 28630072 PMC5563587

[B2] Ahmad I. Rouf S. F. Sun L. Cimdins A. Shafeeq S. Le Guyon S. . (2016). BcsZ inhibits biofilm phenotypes and promotes virulence by blocking cellulose production in Salmonella enterica serovar Typhimurium. Microb. Cell Fact. 15, 177. doi: 10.1186/s12934-016-0576-6, PMID: 27756305 PMC5070118

[B3] Allesen-Holm M. Barken K. B. Yang L. Klausen M. Webb J. S. Kjelleberg S. . (2006). A characterization of DNA release in Pseudomonas aeruginosa cultures and biofilms. Mol. Microbiol. 59, 1114–1128. doi: 10.1111/j.1365-2958.2005.05008.x, PMID: 16430688

[B4] Bannu S. M. Lomada D. Gulla S. Chandrasekhar T. Reddanna P. Reddy M. C. (2019). Potential therapeutic applications of C-phycocyanin. Curr. Drug Metab. 20, 967–976. doi: 10.2174/1389200220666191127110857, PMID: 31775595

[B5] Capatina D. Feier B. Hosu O. Tertis M. Cristea C. (2022). Analytical methods for the characterization and diagnosis of infection with Pseudomonas aeruginosa, A critical review. Anal. Chim. Acta 1204, 339696. doi: 10.1016/j.aca.2022.339696, PMID: 35397917

[B6] Chang Q. Wu C. Lin C. Li P. Zhang K. Xu L. . (2018). The Structure of ampG Gene in Pseudomonas aeruginosa and Its Effect on Drug Resistance. Can. J. Infect. Dis. Med. Microbiol. 2018, 7170416. doi: 10.1155/2018/7170416, PMID: 30598711 PMC6287161

[B7] Chatterjee P. Sass G. Swietnicki W. Stevens D. A. (2020). Review of potential pseudomonas weaponry, relevant to the pseudomonas-aspergillus interplay, for the mycology community. J. Fungi (Basel). 6, 81. doi: 10.3390/jof6020081, PMID: 32517271 PMC7345761

[B8] Defoirdt T. (2018). Quorum-sensing systems as targets for antivirulence therapy. Trends Microbiol. 26, 313–328. doi: 10.1016/j.tim.2017.10.005, PMID: 29132819

[B9] Dranseikienė D. Balčiūnaitė-Murzienė G. Karosienė J. Morudov D. Juodžiukynienė N. Hudz N. . (2022). Cyano-Phycocyanin: Mechanisms of Action on Human Skin and Future Perspectives in Medicine. Plants (Basel). 11 (9), 1249. doi: 10.3390/plants11091249, PMID: 35567250 PMC9101960

[B10] Dulcey C. E. Dekimpe V. Fauvelle D. A. Milot S. Groleau M. C. Doucet N. . (2013). The end of an old hypothesis, the pseudomonas signaling molecules 4-hydroxy-2-alkylquinolines derive from fatty acids, not 3-ketofatty acids. Chem. Biol. 20, 1481–1491. doi: 10.1016/j.chembiol.2013.09.021, PMID: 24239007 PMC3877684

[B11] Gao Y. Chen H. Li W. Zhang Y. Luo J. Zhao L. . (2022). Chloroform extracts of Atractylodes chinensis inhibit the adhesion and invasion of Salmonella typhimurium. BioMed. Pharmacother. 154, 113633. doi: 10.1016/j.biopha.2022.113633, PMID: 36063647

[B12] García-Reyes S. Soberón-Chávez G. Cocotl-Yanez M. (2020). The third quorum- sensing system of Pseudomonas aeruginosa, Pseudomonas quinolone signal and the enigmatic PqsE protein. J. Med. Microbiol. 69, 25–34. doi: 10.1099/jmm.0.001116, PMID: 31794380

[B13] Grossman S. Soukarieh F. Richardson W. Liu R. Mashabi A. Emsley J. . (2020). Novel quinazolinone inhibitors of the Pseudomonas aeruginosa quorum sensing transcriptional regulator PqsR. Eur. J. Med. Chem. 208, 112778. doi: 10.1016/j.ejmech.2020.112778, PMID: 32927392 PMC7684530

[B14] Jurado-Martín I. Sainz-Mejías M. McClean S. (2021). Pseudomonas aeruginosa: an audacious pathogen with an adaptable arsenal of virulence factors. Int. J. Mol. Sci. 22, 3128. doi: 10.3390/ijms22063128, PMID: 33803907 PMC8003266

[B15] Khayyat A. N. Abbas H. A. Khayat M. T. Shaldam M. A. Askoura M. Asfour H. Z. . (2021a). Secnidazole is a promising imidazole mitigator of serratia marcescens virulence. Microorganisms. 9, 2333. doi: 10.3390/microorganisms9112333, PMID: 34835458 PMC8617784

[B16] Khayyat A. N. Hegazy W. A. H. Shaldam M. A. Mosbah R. Almalki A. J. Ibrahim T. S. . (2021b). Xylitol inhibits growth and blocks virulence in serratia marcescens. Microorganisms. 9, 1083. doi: 10.3390/microorganisms9051083, PMID: 34070043 PMC8158113

[B17] Lee J. Zhang L. (2015). The hierarchy quorum sensing network in Pseudomonas aeruginosa. Protein Cell. 6, 26–41. doi: 10.1007/s13238-014-0100-x, PMID: 25249263 PMC4286720

[B18] Li H. Li X. Wang Z. Fu Y. Ai Q. Dong Y. . (2015). Autoinducer-2 regulates Pseudomonas aeruginosa PAO1 biofilm formation and virulence production in a dose-dependent manner. BMC Microbiol. 15, 192. doi: 10.1186/s12866-015-0529-y, PMID: 26420312 PMC4588260

[B19] Liu J. Sun L. Zhang H. Shi M. Dahlgren R. A. Wang X. . (2018). Response mechanisms to joint exposure of triclosan and its chlorinated derivatives on zebrafish (Danio rerio) behavior. Chemosphere. 193, 820–832. doi: 10.1016/j.chemosphere.2017.11.106, PMID: 29874755

[B20] Lories B. Belpaire T. E. R. Yssel A. Ramon H. Steenackers H. P. (2020). Agaric acid reduces Salmonella biofilm formation by inhibiting flagellar motility. Biofilm. 2, 100022. doi: 10.1016/j.bioflm.2020.100022, PMID: 33447808 PMC7798450

[B21] Markus V. Paul A. A. Teralı K. Özer N. Marks R. S. Golberg K. . (2023). Conversations in the gut: the role of quorum sensing in normobiosis. Int. J. Mol. Sci. 24, 3722. doi: 10.3390/ijms24043722, PMID: 36835135 PMC9963693

[B22] Meng X. Ahator S. D. Zhang L. H. (2020). Molecular mechanisms of phosphate stress activation of pseudomonas aeruginosa quorum sensing systems. mSphere. 5, e00119–e00120. doi: 10.1128/mSphere.00119-20, PMID: 32188749 PMC7082139

[B23] Motta J. P. Wallace J. L. Buret A. G. Deraison C. Vergnolle N. (2021). Gastrointestinal biofilms in health and disease. Nat. Rev. Gastroenterol. Hepatol. 18, 314–334. doi: 10.1038/s41575-020-00397-y, PMID: 33510461

[B24] Nihal B. Gupta N. V. Gowda D. V. Mariapan M. (2018). Formulation and development of topical anti acne formulation of spirulina extract. Int. J. Appl. Pharmaceutics. 10, 229. doi: 10.22159/ijap.2018v10i6.26334

[B25] Pan R. Lu R. Zhang Y. Zhu M. Zhu W. Yang R. . (2015). Spirulina phycocyanin induces differential protein expression and apoptosis in SKOV-3 cells. Int. J. Biol. Macromol. 81, 951–959. doi: 10.1016/j.ijbiomac.2015.09.039, PMID: 26410814

[B26] Rashid M. H. Kornberg A. (2000). Inorganic polyphosphate is needed for swimming, swarming, and twitching motilities of Pseudomonas aeruginosa. Proc. Natl. Acad. Sci. U.S.A. 97, 4885–4890. doi: 10.1073/pnas.060030097, PMID: 10758151 PMC18327

[B27] Rashmi M. Meena H. Meena C. Kushveer J. S. Busi S. Murali A. . (2018). Anti-quorum sensing and antibiofilm potential of Alternaria alternata, a foliar endophyte of Carica papaya, evidenced by QS assays and in-silico analysis. Fungal Biol. 122, 998–1012. doi: 10.1016/j.funbio.2018.07.003, PMID: 30227935

[B28] Rather M. A. Gupta K. Mandal M. (2021). Inhibition of biofilm and quorum sensing-regulated virulence factors in Pseudomonas aeruginosa by Cuphea carthagenensis (Jacq.) J. F. Macbr. Leaf extract: An *in vitro* study. J. Ethnopharmacol. 269, 113699. doi: 10.1016/j.jep.2020.113699, PMID: 33340600

[B29] Saeki E. K. Kobayashi R. K. T. Nakazato G. (2020). Quorum sensing system: Target to control the spread of bacterial infections. Microb. Pathog. 142, 104068. doi: 10.1016/j.micpath.2020.104068, PMID: 32061914

[B30] Sanya D. R. A. Onésime D. Vizzarro G. Jacquier N. (2023). Recent advances in therapeutic targets identification and development of treatment strategies towards Pseudomonas aeruginosa infections. BMC Microbiol. 23, 86. doi: 10.1186/s12866-023-02832-x, PMID: 36991325 PMC10060139

[B31] Schertzer J. W. Brown S. A. Whiteley M. (2010). Oxygen levels rapidly modulate Pseudomonas aeruginosa social behaviours *via* substrate limitation of PqsH. Mol. Microbiol. 77, 1527–1538. doi: 10.1111/j.1365-2958.2010.07303.x, PMID: 20662781 PMC3098721

[B32] Sousa A. M. Pereira M. O. (2014). Pseudomonas aeruginosa Diversification during Infection Development in Cystic Fibrosis Lungs-A Review. Pathogens. 3, 680–703. doi: 10.3390/pathogens3030680, PMID: 25438018 PMC4243435

[B33] Szabó S. Feier B. Capatina D. Tertis M. Cristea C. Popa A. (2022). An overview of healthcare associated infections and their detection methods caused by pathogen bacteria in Romania and Europe. J. Clin. Med. 11, 3204. doi: 10.3390/jcm11113204, PMID: 35683591 PMC9181229

[B34] Tang H. Yang D. Zhu L. Shi F. Ye G. Guo H. . (2022). Paeonol interferes with quorum-sensing in pseudomonas aeruginosa and modulates inflammatory responses *in vitro* and *in vivo*. Front. Immunol. 13, 896874. doi: 10.3389/fimmu.2022.896874, PMID: 35686124 PMC9170885

[B35] Vadakkan K. Ngangbam A. K. Sathishkumar K. Rumjit N. P. Cheruvathur M. K. (2024). A review of chemical signaling pathways in the quorum sensing circuit of Pseudomonas aeruginosa. Int. J. Biol. Macromol. 254, 127861. doi: 10.1016/j.ijbiomac.2023.127861, PMID: 37939761

[B36] Venugopal V. C. Thakur A. Chennabasappa L. K. Mishra G. Singh K. Rathee P. . (2020). Phycocyanin Extracted from Oscillatoria minima Shows Antimicrobial, Algicidal, and Antiradical Activities: In silico and *In vitro* Analysis. Antiinflamm Antiallergy Agents Med. Chem. 19, 240–253., PMID: 30950358 10.2174/1871523018666190405114524 PMC7499352

[B37] World Health Organization (2017). WHO Publishes List of Bacteria for Which New Antibiotics Are Urgently Needed. Available online at: https://www.who.int/news/item/27-02-2017-who-publishes-list-of-bacteria-for-which-new-antibiotics-are-urgentlyneeded (Accessed February 27, 2017).

[B38] Xu X. Yu H. Zhang D. Xiong J. Qiu J. Xin R. . (2016). Role of ppGpp in Pseudomonas aeruginosa acute pulmonary infection and virulence regulation. Microbiol. Res. 192, 84–95. doi: 10.1016/j.micres.2016.06.005, PMID: 27664726

[B39] Yang D. Hao S. Zhao L. Shi F. Ye G. Zou Y. . (2021). Paeonol attenuates quorum-sensing regulated virulence and biofilm formation in pseudomonas aeruginosa. Front. Microbiol. 12, 692474. doi: 10.3389/fmicb.2021.692474, PMID: 34421847 PMC8371487

[B40] Ying J. Xu J. Shen L. Mao Z. Liang J. Lin S. . (2017). The effect of sodium fluoride on cell apoptosis and the mechanism of human lung BEAS-2B cells *in vitro*. Biol. Trace Elem Res. 179, 59–69. doi: 10.1007/s12011-017-0937-y, PMID: 28111709

[B41] Yu H. He X. Xie W. Xiong J. Sheng H. Guo S. . (2014). Elastase LasB of Pseudomonas aeruginosa promotes biofilm formation partly through rhamnolipid-mediated regulation. Can. J. Microbiol. 60, 227–235. doi: 10.1139/cjm-2013-0667, PMID: 24693981

[B42] Zhang L. Kong D. Huang J. Wang Q. Shao L. (2022). The therapeutic effect and the possible mechanism of C-phycocyanin in lipopolysaccharide and seawater-induced acute lung injury. Drug Des. Devel Ther. 16, 1025–1040. doi: 10.2147/DDDT.S347772, PMID: 35418745 PMC8995161

