# Supplementary material for: The role of cyano-phycocyanin as a quorum sensing inhibitor to attenuate Pseudomonas aeruginosa virulence
Source: Front Cell Infect Microbiol. 2025 Nov 19;15:1624927. doi: 10.3389/fcimb.2025.1624927 (PMC12672448; doi:10.3389/fcimb.2025.1624927)
Supplement: Supplementary file 1 [file Table1.docx]

**Supplementary** **Table S1 Primer Sequences and Product Sizes**

| **Gene** | **Nucleotide sequence (5'-3')** | **Product size (bp)** |
| --- | --- | --- |
| *chiC* | F: CTGGGAGTTCCGCAAGCGTTAC | 268 |
|  | R: ATCGGTGGCGGTGACGAAATAG |  |
| *exoS* | F: GACGCAAGCCCGGAACT | 184 |
|  | R: CAGGCTGTCTGCCCAGGTAC |  |
| *exsA* | F: GATGCTCGCCTGCCTGAA | 179 |
|  | R: CGAACTCGCGGGAGAAGT |  |
| *flgF* | F: CGGAGGAAAAGGTGGAGATCG | 111 |
|  | R: GGTTCGGGTTGACCAGTTTGA |  |
| *fliE* | F: TCAATCGTCTGATGCTGGAAA | 144 |
|  | R: GCTGGGTCTCGTTCACCTTGT |  |
| *lasA* | F: CTGTGGATGCTCAAGGACTAC | 133 |
|  | R: AACTGGTCTTGCCGATGG |  |
| *lasB* | F: AACCGTGCGTTCTACCTGTT | 94 |
|  | R: CGGTCCAGTAGTAGCGGTTG |  |
| *lasI* | F: GGCTGGGACGTTAGTGTCAT | 104 |
|  | R: AAAACCTGGGCTTCAGGAGT |  |
| *lasR* | F: ACGCTCAAGTGGAAAATTGG | 111 |
|  | R: TCGTAGTCCTGGCTGTCCTT |  |
| *lecA* | F: GAAAGGTGAGGTTCTGGCTA | 85 |
|  | R: GGCGACGATGGTAATGAC |  |
| *pelF* | F: GACTTTCTCCACAGCAAG | 89 |
|  | R: CAGAAGTAATTGACGAAGGA |  |
| *pgsR* | F: TCGTTCTGCGATACGGTGAG | 168 |
|  | R: GCACTGGTTGAAGCGGGAG |  |
| *phzA* | F: AACGGTCAGCGGTACAGGGAAAC | 125 |
|  | R: ACGAACAGGCTGTGCCGCTGTAAC |  |
| *popB* | F: GCGCTTCGACGCTGTTGT | 185 |
|  | R: TTCTTCCGACTCCCTGATCTTCT |  |
| *qseB* | F: GCAACCAACTGGAGCAGAGCC | 81 |
|  | R: GCAGGTGGTGGACGTGGACTT |  |
| *pslB* | F: CAACGAATCCACCTTCATCC | 95 |
|  | R: ACTCGCCGCTCTGTACCTC |  |
| *rhlI* | F: AAGGACGTCTTCGCCTACCT | 130 |
|  | R: GCAGGCTGGACCAGAATATC |  |
| *rhlR* | F: CATCCGATGCTGATGTCCAACC | 101 |
|  | R: ATGATGGCGATTTCCCCGGAAC |  |
| *pqsA* | F: ACCTGACCGAGGTTCTGTTC | 114 |
|  | R: TGGCCTGGGAGAGAATGTAG |  |
| *pqsR* | F: CTGATCTGCCGGTAATTGG | 143 |
|  | R: ATCGACGAGGAACTGAAGA |  |
| *vasG* | F: CCGAGTTCCAGGAGAAGCACA | 125 |
|  | R: CAGCAATATCACCGAATAGGGC |  |
